# Podoplanin expression in cancer-associated fibroblasts enhances tumor progression of invasive ductal carcinoma of the pancreas

**DOI:** 10.1186/1476-4598-12-168

**Published:** 2013-12-20

**Authors:** Koji Shindo, Shinichi Aishima, Kenoki Ohuchida, Kenji Fujiwara, Minoru Fujino, Yusuke Mizuuchi, Masami Hattori, Kazuhiro Mizumoto, Masao Tanaka, Yoshinao Oda

**Affiliations:** 1 Department of Anatomic Pathology, Graduate School of Medical Sciences, Kyushu University, 3-1-1 Maidashi, Fukuoka 812-8582, Japan; 2 Department of Surgery and Oncology, Graduate School of Medical Sciences, Kyushu University, 3-1-1 Maidashi, Fukuoka 812-8582, Japan; 3 Advanced Medical Initiatives, Graduate School of Medical Sciences, Kyushu University, 3-1-1 Maidashi, Fukuoka 812-8582, Japan; 4 Kyushu University Hospital Cancer Center, Fukuoka 812-8582, Japan; 5 Research Fellow of Japan Society for the Promotion of Science, Tokyo, Japan

**Keywords:** Podoplanin, Cancer-associated fibroblast, Pancreatic cancer

## Abstract

**Background:**

Interactions between cancer cells and surrounding cancer-associated fibroblasts (CAFs) play an important role in cancer progression. Invasive ductal carcinoma (IDC) of the pancreas is characterized by abundant fibrous connective tissue called desmoplasia. Podoplanin (PDPN) is a lymphatic vessel marker (D2-40), and expression of PDPN by stromal CAFs has been reported to be a prognostic indicator in various types of cancer.

**Methods:**

Expression of PDPN in pancreatic IDCs was assessed by immunohistochemical examination in 105 patients who underwent pancreatic resection. Primary CAFs were established from pancreatic cancer tissue obtained by surgery. Quantitative reverse transcription-polymerase chain reaction and flow cytometric analysis were performed to investigate PDPN expression in CAFs. We sorted CAFs according to PDPN expression, and analyzed the functional differences between PDPN+ CAFs and PDPN– CAFs using indirect co-culture with pancreatic cancer cell lines. We also investigated the culture conditions to regulate PDPN expression in CAFs.

**Results:**

PDPN expression in stromal fibroblasts was associated with lymphatic vessel invasion (*P* = 0.0461), vascular invasion (*P* = 0.0101), tumor size ≥3 cm (*P* = 0.0038), histological grade (*P* = 0.0344), Union for International Cancer Control classification T stage (*P* = 0.029), and shorter survival time (*P* < 0.0001). Primary CAFs showed heterogeneous PDPN expression *in vitro*. Moreover, migration and invasion of pancreatic cancer cell lines (PANC-1 and SUIT-2) were associated with PDPN expression in CAFs (*P* < 0.01) and expression of CD10, matrix metalloproteinase (MMP) 2, and MMP3. In cultured CAFs, PDPN positivity changed over time under several conditions including co-culture with cancer cells, different culture media, and addition of growth factor.

**Conclusions:**

PDPN-expressing CAFs enhance the progression of pancreatic IDC, and a high ratio of PDPN-expressing CAFs is an independent predictor of poor outcome. Understanding the regulation of the tumor microenvironment is an important step towards developing new therapeutic strategies.

## Background

Pancreatic cancer is one of the most lethal types of cancer. Improvements in the survival of pancreatic cancer patients have been minimal, and the 5-year survival rate remains low [1]. The poor prognosis is related to the difficulty of early diagnosis because of the absence of symptoms, and the lack of effective non-operative treatment modalities such as chemotherapy and radiotherapy [2]. Invasive pancreatic cancer tissue includes cancer, inflammatory, and fibroblastic cells, which interact with each other to create the local microenvironment. Fibroblasts recruited by the cancer tissue are called cancer-associated fibroblasts (CAFs), and play an important role in cancer progression [3,4]. The poor prognosis of pancreatic adenocarcinoma is partially due to the fibroblastic reaction called desmoplasia, which induces a hypovascular environment [5], inefficiency of drug delivery, and tumor-stromal interactions such as secretion of growth factors [6].

In 1998, pancreatic stellate cells (PSCs) were identified in the pancreas [7,8]. In the normal pancreas, PSCs are located close to the acinar cells, and retain abundant vitamin A-containing lipid droplets in their cytoplasm as a quiescent phenotype [9]. PSCs can obtain a myofibroblast-like morphology and immunoreactivity for alpha-smooth muscle actin (α-SMA) by inflammatory stimulation or signals from cancer cells, such as interleukin (IL)-1, IL-6, tumor necrosis factor-alpha, platelet-derived growth factor, transforming growth factor (TGF)-β1, and activin A [10]. Activated PSCs produce abundant extracellular matrix (ECM), leading to cancer progression [9,11]. In addition, activated PSCs harvested by the outgrowth method have characteristics similar to those of CAFs. Recently, CAFs have gained attention as a potential therapeutic target [12,13].

Podoplanin (PDPN) is a 38–44 kDa O-glycosylated transmembrane glycoprotein that is selectively expressed by lymphatic endothelial cells [14]. PDPN is also expressed by normal kidney podocytes [15], alveolar type I cells [16], basal epidermal keratinocytes [17], and mesothelial cells [18,19]. Several types of cancer may also express PDPN, such as squamous cell carcinomas [20,21], soft tissue tumors [22], and brain tumors [23]. PDPN-expressing cancer cells have enhanced malignant potential due to enhancement of platelet aggregation, which promotes metastasis [24,25], alteration of cell morphology and motility [26,27], and epithelial-mesenchymal transition [28]. Stromal fibroblasts surrounding cancer cells may also express PDPN [29-36]. The presence of PDPN-expressing fibroblasts has been reported to be a prognostic indicator in several types of cancer, but outcomes vary according to the type of cancer [30,33-36]. Understanding the molecular mechanisms of PDPN expression is important for the development of new therapeutic strategies for the treatment of malignant tumors with PDPN-positive fibroblasts.

In this study, we examined PDPN expression in invasive ductal carcinoma (IDC) of the human pancreas using immunohistochemical methods, and investigated the functional roles of PDPN-expressing CAFs established from pancreatic IDCs by cell sorting. CD10+ PSCs were previously found to enhance the progression of pancreatic cancer in a similar manner to CAFs, depending on matrix metalloproteinase (MMP) 3 secretion [13]. In this study, we found that PDPN+ and PDPN– CAFs had functional differences associated with their expression of CD10, MMP3, and MMP2. We also found that PDPN expression in CAFs was affected by both cancer cell-stromal interactions and environmental conditions.

## Results

### Correlations between PDPN expression in fibroblasts and clinicopathologic characteristics

**Figure 1:**
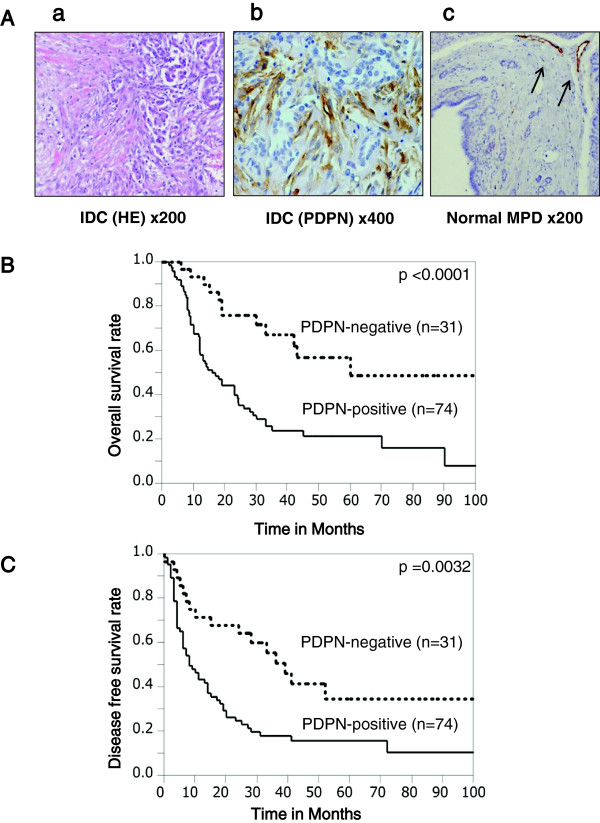
**Immunohistochemical staining for PDPN in IDC of the pancreas. (A-a)** Pancreatic IDCs consisted of cancer cells and stromal cells such as CAFs. **(A-b)** CAFs around the cancer cells were positive for PDPN. **(A-c)** In the normal main pancreatic duct, stromal cells were rarely positive for PDPN. The arrow indicates lymphatic vessels as a positive control. **(B, C)** Stromal PDPN expression was associated with a worse overall survival rate **(B)** and disease-free survival rate **(C)** by Kaplan-Meier survival analysis.

Numerous fibroblasts were observed in the area of cancerous invasion (Figure 1A-a), and PDPN+ fibroblasts were observed close to the tumor cells (Figure 1A-b). In normal pancreatic tissue including the main pancreatic duct, PDPN+ fibroblasts were rarely observed (Figure 1A-c). PDPN+ fibroblasts (≥30%) were observed in 70.5% (74/105) of pancreatic IDCs, but no PDPN+ cancer cells were observed. Patients in the PDPN+ group had more frequent lymphatic invasion (*P* = 0.0461), vascular invasion (*P* = 0.0101), tumor size ≥3 cm (*P* = 0.0038), G3 grade tumor (*P* = 0.0344), and Union for International Cancer Control (UICC) grade pT3 or higher (*P* = 0.029) than patients in the PDPN– group (Table 1). These results suggest that PDPN+ stromal fibroblasts are associated with tumor progression and the invasiveness of cancer cells.

**Table 1:** Relationships between PDPN expression and clinicopathologic factors

**Characteristics**		**PDPN negative, n = 31 (29.5%)**	**PDPN positive, n = 74 (70.5%)**	**p Value**
Lymphatic invasion	No	11(35.5)	13(17.6)	0.0461^*^
	Yes	20(64.5)	61(82.4)	
Vascular invasion	No	17(54.8)	21(28.4)	0.0101^*^
	Yes	14(45.2)	53(71.6)	
Tumor size	<3 cm	23(74.2)	32(43.2)	0.0038^*^
	≥3 cm	8(25.8)	42(56.8)	
Histologic Grade	G1/G2	20(64.5)	31(41.9)	0.0344^*^
	G3	11(35.5)	43(58.1)	
UICC	pT1/pT2	8(25.8)	7(9.5)	0.0290^*^
T category	pT3/pT4	23(74.2)	67(90.5)	
UICC	pN0	14(45.2)	20(27.0)	0.0701
N category	pN1	17(54.8)	54(73.0)	
UICC stage	I	7(22.6)	5(6.8)	0.0642
	II	23(74.2)	65(87.8)	
	III/IV	1(3.2)	4(5.4)	

Significant differences between samples are indicated as *P < 0.05. UICC, Union for International Cancer Control.

### Stromal PDPN expression is independently associated with shorter survival time

PDPN+ stromal fibroblasts were associated with shorter patient survival and disease-free survival times (Figure 1B,C). The median survival times for PDPN+ and PDPN– cases were 14 and 33 months, respectively. Multivariate survival analysis based on the Cox proportional hazards model including all parameters found to be significant on univariate analyses (data not shown), including PDPN positivity, lymphatic invasion, vascular invasion, tumor size ≥3 cm, G3 tumor, UICC grade pT3/4, UICC grade pN1, and stage III/IV (Table 2) [37], found that PDPN positivity (relative risk 2.598, *P* = 0.0030) and UICC N1 category (*P* = 0.0302) were independent markers of poor prognosis (Table 2) [37].

**Table 2:** Multivariate survival analysis (Cox regression model) of conventional prognostic factors and PDPN expression

	**Relative Risk**	**95% Confidence interval**	**p Value**
PDPN positivity	2.598	1.363-5.390	0.003^*^
Lymphatic invasion	1.483	0.643-3.892	0.3694
Vascular invasion	1.389	0.736-2.720	0.3149
Tumor size	1.311	0.745-2.322	0.3474
Histological Grade	1.151	0.676-1.988	0.6061
UICC T category	0.245	0.067-1.580	0.1198
UICC N category	2.07	1.069-4.282	0.0302^*^
UICC Stage			0.1550

NOTE. The relative risk of the UICC stage is not shown because it is comprised of two parameters. Significant differences are indicated as *P < 0.05. UICC, Union for International Cancer Control, 7^th^ edition.

### CAFs have heterogeneous PDPN expression

**Figure 2:**
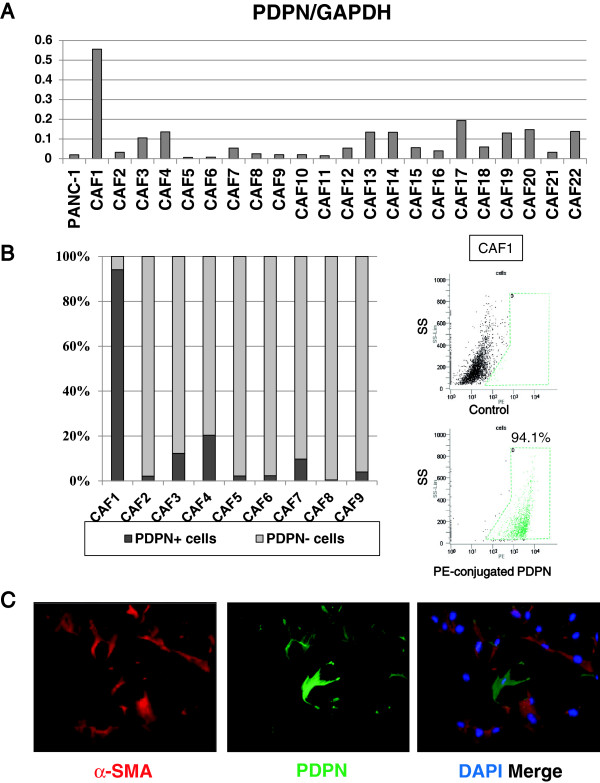
**PDPN expression was heterogeneous in CAFs. (A)** qRT-PCR showed variable PDPN expression in CAFs. The pancreatic cancer cell line PANC-1 expressed less PDPN mRNA than CAFs. **(B)** Flow cytometry showed 0.4–94% positivity rates for PDPN expression. Representative flow cytometry data for PDPN expression in CAF1 cells are shown (right). **(C)** Immunofluorescence staining for PDPN (green) and α-SMA (red) in CAF4. CAFs had spindle-shaped or stellate-like morphology and expressed α-SMA and PDPN. Original magnification × 100.

We investigated PDPN mRNA expression in 22 CAF cultures that were numbered from 1 to 22 by quantitative reverse transcription-polymerase chain reaction (qRT-PCR) (Figure 2A). Primary CAF cultures expressed various levels of PDPN mRNA, and CAF1 cells showed significantly high expression. The pancreatic cancer cell line PANC-1 did not express PDPN mRNA. Flow cytometric analysis showed that cultured CAFs exhibited various positivity rates (0.4–94%) for PDPN in accordance with the results of qRT-PCR (Figure 2B). We confirmed that PDPN expression had not been affected by proteolysis during trypsin/EDTA treatment using flow cytometric analysis (Additional file 1: Figure S1A), and immunocytochemical staining of CAFs (Additional file 1: Figure S1B). All the primary cultured CAFs (CAF1, CAF2, CAF3, and CAF4) were positive for fibroblast activation protein-alpha (FAP) in most cells (Additional file 1: Figure S1C). Immunofluorescence staining showed CAFs as spindle-shaped and stellate-like cells, and almost all cells were stained for α-SMA and partly stained for PDPN (Figure 2C). CAFs are therefore heterogeneous in terms of PDPN expression.

### CAFs enhance the invasive potential of pancreatic cancer cells

**Figure 3:**
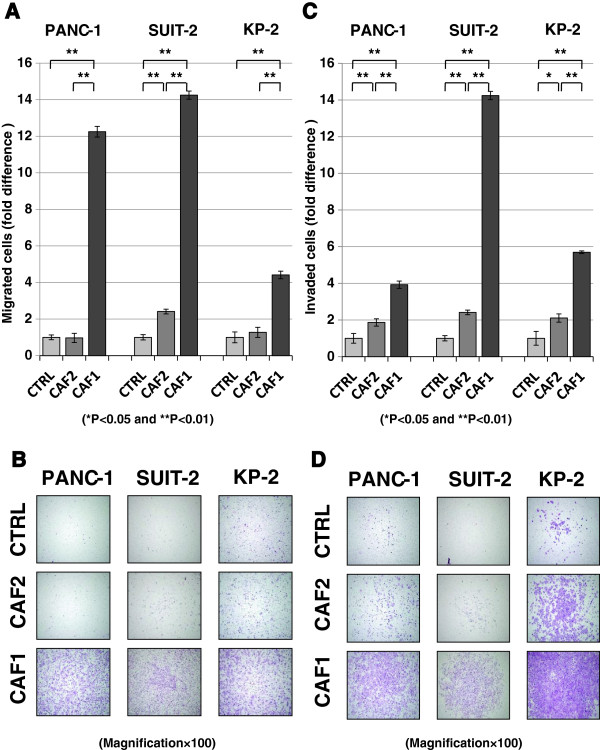
**Effects of CAFs (CAF1 and CAF2 cells) on the invasive potential of pancreatic cancer cells.** CAF1 (high PDPN-expressing cells) promoted the migration **(A, B)** and invasion **(C, D)** of PANC-1, SUIT-2, and KP-2 cells compared with CAF2 (PDPN– cells). Significant differences between sample means are indicated as *P < 0.05, **P < 0.01. **(B, D)** Representative photomicrographs of migrating **(B)** and invading **(D)** cancer cells co-cultured with CAFs (hematoxylin and eosin staining; original magnification × 40).

To investigate the effects of CAFs on the invasiveness of pancreatic cancer cells, we used a transwell co-culture system as described previously [13]. Fujita et al. [38] reported that the proliferation of pancreatic cancer cells was not enhanced by the conditions of an indirect co-culture system. We confirmed those findings, and assumed that the increase in invasiveness of cancer cells was not accelerated by the proliferation of cancer cells. PANC-1, SUIT-2, and KP2 pancreatic cancer cells were co-cultured with CAF1 (high PDPN-expressing cells) or CAF2 (PDPN– cells) in the transwell system. Pancreatic cancer cells strongly migrated (Figure 3A,B) and invaded (Figure 3C,D) when co-cultured with CAF1 cells. On the other hand, CAF2 cells had a lesser effect on migration and invasion of co-cultured cancer cells. Therefore, we hypothesized that the differences in the invasive potential of cancer cells were due to PDPN expression in CAFs.

### PDPN+ CAFs enhance the invasive potential of pancreatic cancer cells more effectively than PDPN– CAFs

**Figure 4:**
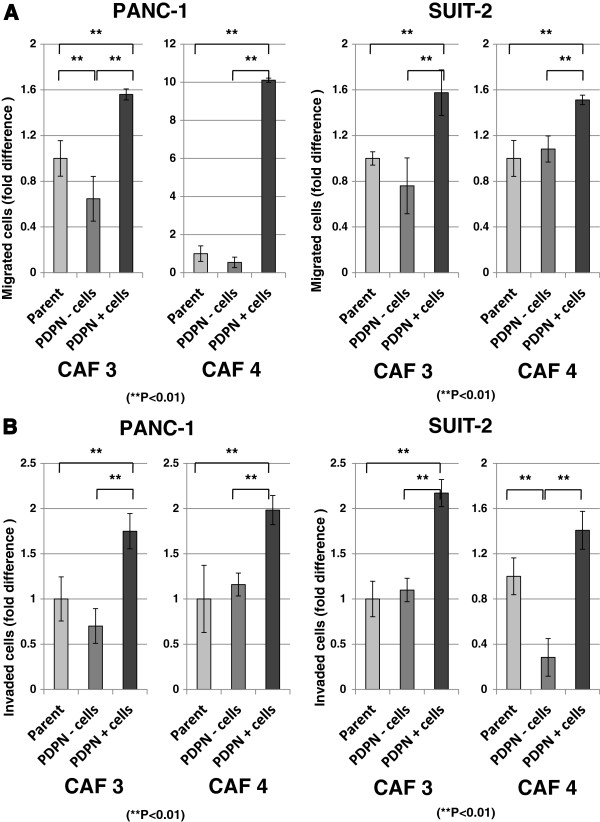
**Effects of sorted CAFs on the invasiveness of pancreatic cancer cells.** PDPN+ CAFs promoted the migration **(A)** and invasion **(B)** of PANC-1 and SUIT-2 cells compared with PDPN– CAFs. The *P* values for the migration assays were: *P* = 0.0001 (PANC-1 with CAF3), *P* = 0.0003 (PANC-1 with CAF4), *P* = 0.0034 (SUIT-2 with CAF3), and *P* = 0.0058 (SUIT-2 with CAF4). The *P* values for the invasion assays were: *P* = 0.0013 (PANC-1 with CAF3), *P* = 0.0022 (PANC-1 with CAF4), *P* = 0.0065 (SUIT-2 with CAF3), and *P* = 0.0013 (SUIT-2 with CAF4). Significant differences between sample means are indicated as **P < 0.01.

To clarify the differences in enhancement of invasive potential between PDPN+ and PDPN– CAFs, we sorted CAFs by magnetic-activated cell sorting (Additional file 2: Figure S2A). The level of PDPN mRNA expression in PDPN+ and PDPN– CAFs derived from CAF3 and CAF4 cells was consistent with the level of PDPN expression found by flow cytometry (Additional file 2: Figure S2B). We investigated the effects of these two populations on the invasiveness of PANC-1 and SUIT-2 cells using migration and invasion assays in a co-culture system. PDPN+ CAF3 and CAF4 cells enhanced the migration (Figure 4A) and invasion (Figure 4B) of PANC-1 and SUIT-2 cells more strongly than PDPN– CAFs (*P* < 0.01).

### Knockdown of PDPN in CAFs has no effect on the enhancement of invasiveness of pancreatic cancer cells

PDPN is a known transmembrane protein, but not a secreted protein. Despite no detection of PDPN in culture supernatants of CAFs (data not shown), PDPN+ CAFs had a promoting effect on cancer cell invasion in the indirect co-culture system. We compared CAF1 cells with and without PDPN knockdown by small interfering RNA (siRNA) (Additional file 3: Figure S3A,B) to clarify the functional role of PDPN. There were no changes in migration (Additional file 3: Figure S3C) or invasion (Additional file 3: Figure S3D) of PANC-1 and SUIT-2 cells when they were co-cultured with PDPN-knockdown CAF1 cells. These data suggest that PDPN itself has no effect on the invasion and migration of pancreatic cancer cells, whereas PDPN+ CAFs promote these features.

### Differences in cancer cell invasion-related gene expression between PDPN+ and PDPN– CAFs

**Figure 5:**
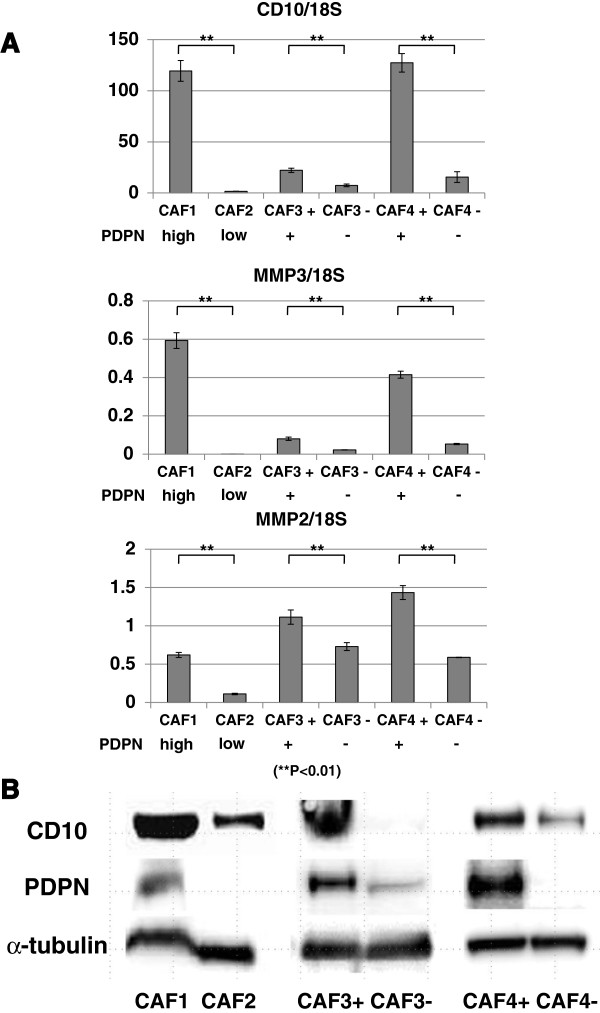
**Differences between PDPN+ and PDPN– CAFs. (A)** qRT-PCR analyses showed that the levels of PDPN mRNA expression in CAFs were significantly correlated with the levels of CD10, MMP2, and MMP3 mRNA expression. Significant differences between sample means are indicated as **P < 0.01. **(B)** Western blot analysis showed that PDPN protein expression in CAFs was also associated with CD10 protein expression.

To detect differences between PDPN+ and PDPN– CAFs, we analyzed the mRNA and protein from various CAFs (CAF1, CAF2, and CAF3 and CAF4 cells sorted by PDPN expression). CAF1 cells and PDPN+ CAFs expressed more CD10, MMP3, and MMP2 than CAF2 cells and PDPN– CAFs (Figure 5A). CD10 is a cell surface metalloproteinase, and MMPs promote cancer cell invasion by degrading structural ECM proteins [39,40]. In particular, CD10+ PSCs have been reported to promote cancer-stromal interactions by secreting high levels of MMP3 [13]. The level of PDPN protein expression in CAFs was correlated with that of CD10 expression (Figure 5B), which was also the trend observed in qRT-PCR analysis. These findings suggest that PDPN+ CAFs in pancreatic cancer play a pivotal role in increasing the invasive potential of cancer cells.

### The number of PDPN+ CAFs is increased through cancer-stromal interactions

**Figure 6:**
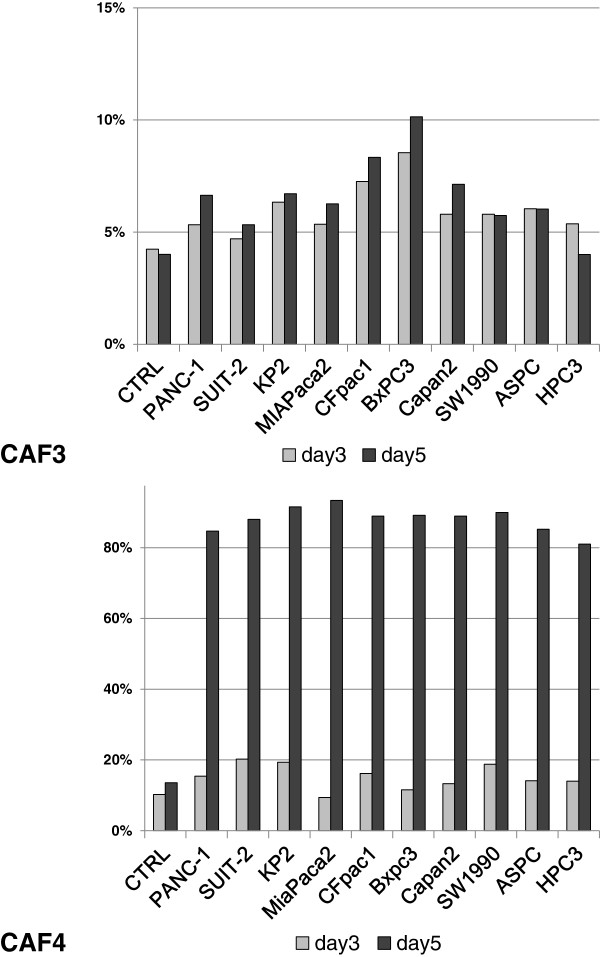
**Percentage of PDPN+ cells among CAFs indirectly co-cultured with pancreatic cancer cells.** CAFs were co-cultured with the indicated pancreatic cancer cell lines for several days, and then the percentage of PDPN+ CAFs was assessed by flow cytometry.

We investigated the effects of cancer cells on PDPN expression in CAFs using an indirect co-culture system. When co-cultured with a variety of pancreatic cancer cell lines in Dulbecco’s modified Eagle’s medium (DMEM) / 2% fetal bovine serum (FBS), the populations of PDPN-expressing CAFs increased in a time-dependent manner, especially CAF4 on day 5, compared with CAF monocultures (Figure 6). The numbers of CAF4 were almost same on days 3 and 5, indicating a possible change in the expression pattern from PDPN– to PDPN+ .

### The number of PDPN+ CAFs is affected by the culture conditions

**Figure 7:**
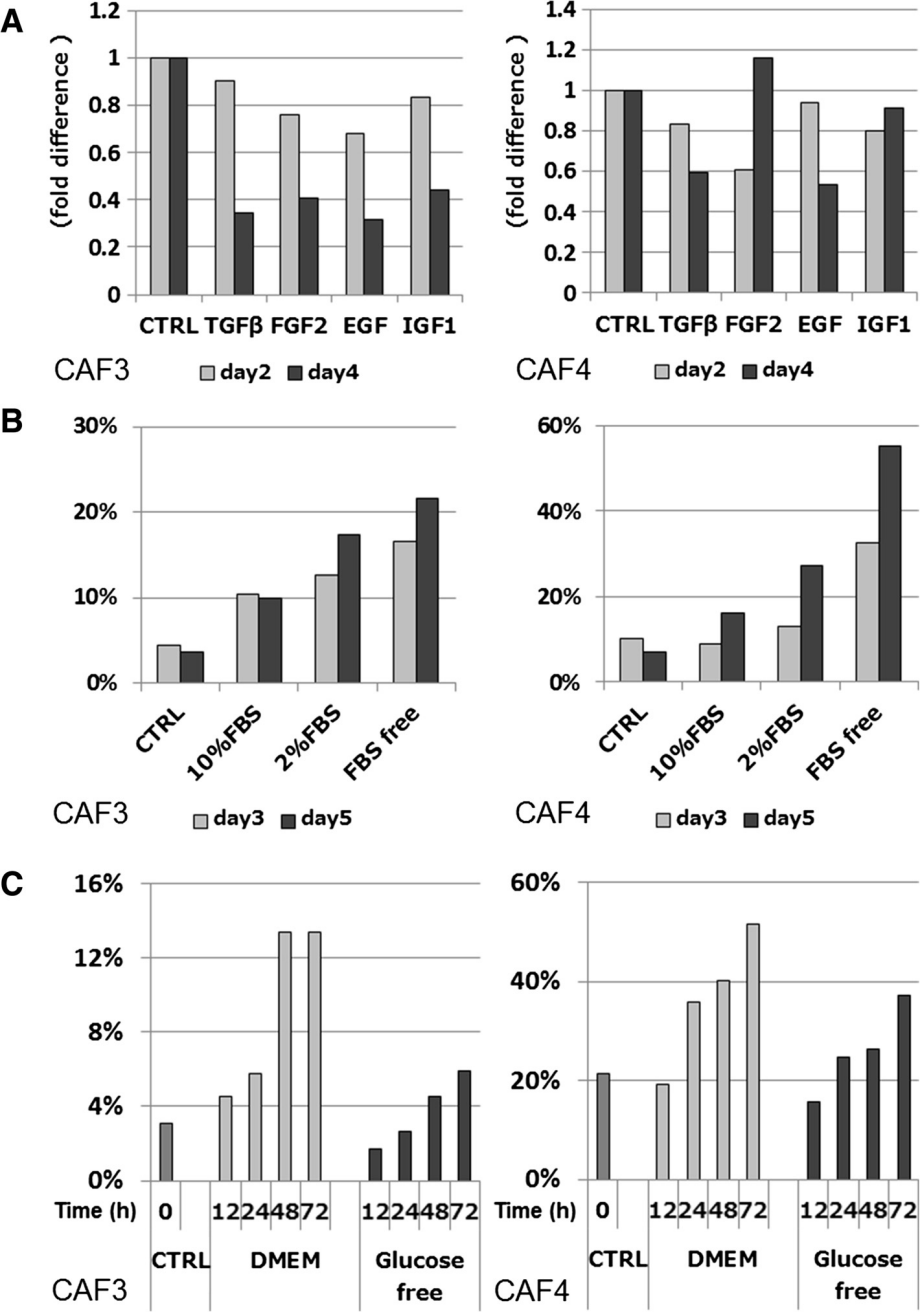
**Populations of PDPN+ cells among CAFs affected by environmental conditions. (A)** The percentage of PDPN+ cells among CAFs changed in a time-dependent manner by culturing with DMEM-containing growth factors. **(B)** Populations of PDPN+ cells among CAFs changed by the addition of FBS in concentration- and time-dependent manners. The control (CTRL) condition consisted of DMEM containing 10% FBS and medium changes every day. **(C)** Populations of PDPN+ cells in cultures of serum-free DMEM increased more rapidly than those in cultures of glucose- and serum-free DMEM.

In co-cultures, all cancer cell lines significantly increased PDPN expression in CAF4 cells on day 5. Therefore, we hypothesized that PDPN expression was not only affected by specific signals, but also by environmental conditions. Wicki et al. [27] reported that PDPN expression in the breast cancer cell line MCF7 is up-regulated by stimulating growth factors such as TGF-β, fibroblast growth factor (FGF)-2, and epidermal growth factor (EGF). However, when we added the growth factors TGF-β1, FGF-2, insulin-like growth factor (IGF)-1, and EGF to serum-free DMEM, PDPN+ populations among CAF3 and CAF4 cells mostly decreased in a time-dependent manner (Figure 7A). In CAF4 cell cultures, however, addition of FGF-2 or IGF-1 had a lesser effect on PDPN+ populations (Figure 7A). Next, we investigated the effects of added FBS on PDPN+ populations. We compared PDPN+ populations among CAF3 and CAF4 cells when cultured in DMEM with or without several concentrations of FBS. Control cells were cultured in DMEM with 10% FBS, and the medium was changed every day. Addition of FBS attenuated the increasing rate of PDPN+ populations, depending on the FBS concentration. However, PDPN+ cells among CAFs increased in a time-dependent manner despite administration of FBS (Figure 7B). In addition, the populations of PDPN+ CAFs cultured in serum-free DMEM increased more significantly than those cultured in glucose- and serum-free DMEM (Figure 7C). These findings suggest that PDPN+ CAFs are affected by culture conditions, and PDPN+ populations increase in a time-dependent manner when cultured in DMEM containing glucose and decrease in high concentrations of FBS and growth factors.

## Discussion

In the present study, PDPN-expressing CAFs were found in IDC tissue of the pancreas. We revealed the significance of these cells in terms of cancer cell invasion, in correlation with shorter patient survival and several biological factors, namely lymphatic and vascular invasion, larger tumor size, histological grade, and UICC T grade [37]. Kitano et al. [33] assessed PDPN-expressing stromal spindle cells in multiple cancer tissues including pancreatic cancer using tissue microarrays. They found a 73% (16/22) positivity rate for PDPN expression in pancreatic cancer. In this study, positive PDPN expression was found in 70.5% (74/105) of cases when we defined the cutoff value in stromal fibroblasts as 30%. We therefore consider this to be an appropriate cutoff value.

In recent clinicopathological studies of other cancer types, PDPN expression in the cancerous stroma was reported to be a prognostic indicator, but the effects on prognosis varied depending on the cancer type. High PDPN expression in fibroblasts is significantly correlated with a poor prognosis in IDC of the breast [34], lung adenocarcinoma [31-33], and ovarian carcinoma [36]. Conversely, PDPN-expressing fibroblasts indicate a favorable outcome in colorectal carcinoma [35] and uterine cervical carcinoma [30].

Some of the biological functions of PDPN in cancer cells have been partially elucidated in several studies, but the biological characteristics of PDPN-expressing stromal fibroblasts are largely unknown. Yamanashi et al. [35] showed increased invasion of colorectal cancer cells when they were co-cultured with fibroblasts with PDPN knockdown by siRNA. However, the interactions between the cells were not described, and the fibroblasts were from a colonic fibroblast cell line (CCD112CoN from 22 weeks of gestation) rather than from cancer tissue.

Knockdown of PDPN by siRNA had no effect on the enhancement of cancer cell invasiveness in our study. However, Hoshino et al. [31] reported that PDPN-expressing CAFs in lung adenocarcinoma promote tumor formation both *in vivo* and *in vitro* using transfection by short hairpin RNA against PDPN expression. According to transfection studies in human MCF7 breast cancer cells, PDPN expression results in morphological changes, induction of migratory phenotypes with a significant decrease in cellular stress fibers, and an increase in filopodia-like protrusions [27]. In our study, PDPN knockdown CAFs did not show any notable changes in morphology compared with parental CAFs (data not shown). The differences in alteration of morphology might therefore be dependent on the cell type.

PSCs were originally identified as the source of fibrosis in chronic pancreatitis [7], and were assumed to be the source of desmoplasia in pancreatic cancers. We used PSCs obtained by the outgrowth method as CAFs, because activated PSCs have a myofibroblast-like shape and are positive for α-SMA and vimentin, which is similar to CAFs. It was therefore very difficult to differentiate between PSCs and CAFs on immunohistochemical staining. The characteristic differences in CAFs originating from different tumors might be explained by them originating from diverse sources, such as resident local fibroblasts, bone marrow-derived progenitor cells, and transdifferentiation of epithelial/endothelial cells by epigenetic transition [41]. Vascular adventitial fibroblasts in lung adenocarcinoma have biological functions similar to those of CAFs, and PDPN is highly expressed in vascular adventitial fibroblasts in association with cancer progression [31]. The differences in biological function of PDPN+ CAFs in diverse cancers might therefore be based on the characteristics of their origins.

With the increasing understanding of the roles of CAFs, various discussions exist regarding their origins, specific markers, and characteristics [42]. Erez et al. [43] reported the differences in proinflammatory genes as signature genes between normal fibroblasts and CAFs in pancreatic ductal adenocarcinoma especially in mice. Cyclooxygenase 2, Chemokine (C-X-C motif) ligand (CXCL) 1, CXCL2, cysteine-rich 61, IL-1β, IL-6, and osteopontin differed in their mRNA expression. When we investigated these signature genes in our CAFs (CAF1, CAF2, CAF3+/-, and CAF4+/-), the results were variable, and were not associated with PDPN expression (data not shown). Several markers for CAFs have been identified, including α-SMA and FAP. In previous studies, FAP-expressing fibroblasts were reported to enhance the cancer cell invasion by producing ECM [44], and have essential functions in maintaining muscle mass and hematopoiesis [45]. Most of the primary cultured CAFs in our study were α-SMA positive [13] and FAP positive.

The interactions among cancer cells, stromal cells such as CAFs, and inflammatory cells create the tumor microenvironment, and remodel the surrounding ECM when cancer cells become invasive. Growth factors also have important effects on adjacent cells in an autocrine and paracrine fashion [11]. In addition, MMPs are known to play important roles in cell migration and degradation of the surrounding ECM [40].

In our laboratory, Fujita *et al.* reported that conditioned medium from PSCs established by the outgrowth method enhanced colony formation of SUIT-2 cells in the same way as co-culture. However, colony formation of MIAPaCa-2 cells was not enhanced by the conditioned medium [46]. Ikenaga *et al.* revealed that CD10-expressing PSCs promoted the invasiveness of cancer cells by secreting MMP3, which was confirmed in the supernatant [13]. Hwang *et al.* also reported that conditioned medium from PSCs stimulated cancer cell proliferation, invasion, and colony formation [3]. The effect of PDPN expression on the conditioned medium of CAFs in this study was unclear. However it is likely that this medium would have similar effects on the invasiveness of cancer cells as co-culture with CAFs, given the differences in CD10 expression and MMP secretion between PDPN-positive and -negative CAFs.

FBS contains high concentrations of embryonic growth-promoting factors, and is widely used as a growth supplement to enhance cell survival and proliferation, although the composition of FBS is not fully understood. We found that PDPN+ CAFs were important modulators of MMP expression. In addition, the reduction in the numbers of PDPN+ CAFs after addition of growth factors or high concentrations of FBS suggests the possibility of negative feedback by growth factors.

## Conclusions

Although the molecular mechanisms of PDPN expression in CAFs are not clear, and CAFs have functional heterogeneity, we found that PDPN+ CAFs play an important role in cancer cell invasion in association with the expression of CD10, MMP2, and MMP3. It is important to develop new therapeutic strategies to target the supportive microenvironment provided by cancer-stromal interactions in pancreatic cancer.

## Methods

### Patients and pancreatic tissue

Pancreatic cancer tissue was obtained from 105 patients who underwent pancreatic resection for IDC of the pancreas at our institution from 1995 to 2011. The clinicopathologic characteristics of the patients are shown in Additional file 4: Table S1. The patients included 70 men and 35 women with a median age of 65 years (range: 43–86 years). Survival was measured from the time of pancreatic resection until death or censor. The follow-up duration ranged from 1 to 137 months, and the median overall survival time was 19 months. Seventy-three patients died during follow-up. The histological diagnosis of the specimens was confirmed according to the criteria of the updated World Health Organization classification [47]. The tumor stage was assessed according to the UICC classification, 7^th^ edition [37]. Lymphatic and vascular invasion were detected by hematoxylin and eosin staining. When necessary, we performed D2-40 (PDPN) staining to determine lymphatic invasion and Elastica van Gieson staining to determine vascular invasion. Patients were divided into groups for statistical analysis as shown in Table 1. We also obtained 20 normal pancreatic tissue samples from intact pancreatic specimens that were resected for solid-pseudopapillary neoplasm or neuroendocrine tumor, as control tissue. This study was approved by the Ethics Committee of Kyushu University (approval number 25–23, 24–222) and was conducted according to the Ethical Guidelines for Human Genome/Gene Research enacted by the Japanese Government and the Helsinki Declaration.

### Cells and culture conditions

Human CAFs were isolated from fresh surgical specimens of pancreatic cancer using the outgrowth method [8]. Primary cultures of CAFs derived from 22 patients with invasive pancreatic cancer were established in our laboratory. The cell type was confirmed by a spindle-shaped morphology and immunofluorescence staining for α-SMA and vimentin [13,48]. Passage 3–8 cells were used for assays. In addition, the following 10 pancreatic cancer cell lines were used: PANC-1, SUIT-2, KP-2, and AsPC-1 (Dr. Iguchi, National Shikoku Cancer Center, Matsuyama, Japan); MIAPaCa-2 (Japanese Cancer Resource Bank, Tokyo, Japan); BxPC-3, Capan-2, CFPAC-1, and SW 1990 (American Type Culture Collection, Manassas, VA); and HPC-3 (Dr. Yasoshima, Sapporo Medical University, Hokkaido, Japan). The lung squamous cancer cell line H157 (Dr. Onimaru, Kyushu University, Fukuoka, Japan) was used as a positive control for PDPN expression [20]. Cells were maintained as described previously [20,49]. In the PDPN induction assay, DMEM (Sigma Chemical Co., St. Louis, MO) or DMEM containing no glucose (Invitrogen, Carlsbad, CA) was used for cell culture. Media were supplemented with 2% or 10% fetal bovine serum (FBS) (Invitrogen), 10 ng/ml TGF-β1 (R&D, Oxon, UK), 50 ng/ml FGF-2 (Sigma, Basel, Switzerland), 100 ng/ml IGF-1 (R&D), and 100 ng/ml EGF (Sigma).

### Immunohistochemical procedures and evaluation

Immunohistochemical staining was conducted as described previously [29]. Formalin-fixed, paraffin-embedded tissue was cut at 4-μm thicknesses and deparaffinized with xylene and ethanol. The endogenous peroxidase activity was blocked by methanol containing 0.3% hydrogen peroxidase. Antigen retrieval was performed by boiling in a microwave oven (citrate buffer, pH 6.0). The sections were incubated overnight at 4°C with primary antibodies against PDPN (413541; mouse monoclonal, D2-40; Nichirei Bioscience, Tokyo, Japan) and α-SMA (A2547; mouse monoclonal, 1:400; Sigma, St Louis, MO). The immune complexs were then visualized using EnVision Detection System (Dako, Glostrup, Denmark) and 3,3′-diaminobenzidine (DAB) Kit (Dako). We performed immunohistochemical staining for PDPN and α-SMA in consecutive sections, and fibroblast-like cells involved with the cancer cells or cancer ducts were α-SMA positive, as shown in a previous report [46]. We confirmed the cell type by a spindle-shaped morphology and α-SMA positivity. PDPN was also expressed by such fibroblast-like cells, and we assumed those cells to be CAFs. All sections were evaluated independently by two investigators without any knowledge of the clinical features. Stromal expression of PDPN was defined as positive when over than 30% of the stromal fibroblasts around neoplastic cells were stained. The stromal cells around normal pancreatic ducts were also evaluated in 20 cases of normal pancreatic tissue. PDPN expression was not observed in any carcinoma cells. Lymphatic vessels stained positive for PDPN without exception, and were used as a positive control.

### Immunocytochemical staining of CAFs

Immunocytochemical staining of CAFs was conducted using a streptavidin-biotin-peroxidase complex method (Histofine; Nichirei, Tokyo, Japan), with primary antibodies against PDPN (413541; mouse monoclonal, D2-40, Nichirei Bioscience). Cultured cells were fixed on culture slides for 20 min in methanol/acetone at 4°C. Endogenous peroxidase activity was blocked by treatment with methanol containing 0.3% hydrogen peroxidase for 20 min. Antigen retrieval was conducted by microwave heating for 20 min with sodium citrate buffer (pH 6.0). After exposure to 10% non-immunized goat serum in PBS for 20 min, sections were incubated with primary antibody at room temperature for 90 min. Subsequent reactions were performed according to the peroxidase-labeled streptavidin-biotin technique using a histofine SAB-PO kit (Nichirei). The reaction products were visualized using diaminobenzidine tetrahydrochloride as a chromogen. Finally, the sections were counterstained with hematoxylin. Harvested CAFs were treated with trypsin/EDTA for 5 min, then centrifuged at 1,600 × *g.* for 5 min and fixed for 3 h by mixing with 5 mL 10% formalin. Cells were centrifuged for 5 min and the supernatant was removed, followed by the addition of 0.5 ml 1% sodium alginate. After centrifugation for a further 5 min, 2 drops of 1 M calcium chloride solution were added. The concretions were embedded in paraffin. Sections were cut at 4 μm and stained with PDPN.

### Real-time qRT-PCR

One-step real-time qRT-PCR with gene-specific priming was performed as described previously [13]. The total RNA was extracted from cultured cells using a High Pure RNA Isolation Kit (Roche Diagnostics, Mannheim, Germany) and DNase I (Roche Diagnostics) treatment according to the manufacturer’s instructions. One-step real-time qRT-PCR was performed using a QuantiTect SYBR Green Reverse Transcription-PCR Kit (Qiagen, Tokyo, Japan) and a CFX96 Touch Real-Time PCR Detection System (Bio-Rad Laboratories, Hercules, CA). Primers for PDPN, CD10, MMP2, MMP3, glyceraldehyde-3-phosphate dehydrogenase (GAPDH), and 18S ribosomal RNA (18SrRNA) were purchased from Takara Bio Inc. (Tokyo, Japan). The primer sequences are listed in Additional file 5: Table S2. Each reaction mixture was first incubated at 50°C for 30 minutes to allow reverse transcription in which first-strand complementary DNA was synthesized by priming total RNA with a gene-specific primer. PCR was initiated by incubation at 95°C for 15 minutes to activate the polymerase, followed by 40 cycles of 95°C for 5 seconds, 60°C for 20 seconds, and 72°C for 30 seconds. The gene expression levels were calculated using a standard curve constructed with total RNA from H157 (a lung squamous cell line), SUIT-2 (a pancreatic cancer cell line), or specific CAFs. The levels of gene expression were normalized to those of GAPDH or 18SrRNA as an internal control and calculated as the ratio of target gene expression to GAPDH or 18SrRNA expression. The quantitative ranges of threshold cycles observed were 15–35 cycles for each of the target genes and 5–25 cycles for GAPDH and 18SrRNA. All samples were run in triplicate, and each sample was analyzed three times. No detectable PCR products were amplified without prior reverse transcription. The accuracy and integrity of the PCR products were confirmed using an Agilent 2100 Bioanalyzer (Agilent Technologies Inc., Palo Alto, CA).

### Immunofluorescence staining

CAFs were plated on glass-bottom dishes (Matsunami, Osaka, Japan) and incubated for 24 hours in DMEM supplemented with 10% FBS. The cells were then fixed with methanol, blocked with 3% bovine serum albumin in PBS, and incubated with mouse monoclonal anti-α-SMA (N1584; 1:50; Dako) or rabbit polyclonal anti-PDPN (sc-134482; 1:50; Santa Cruz Biotechnology, Santa Cruz, CA) antibodies overnight at 4°C. The cells were then incubated with Alexa Fluor 546-conjugated anti-mouse IgG and Alexa Fluor 488-conjugated anti-rabbit IgG (Molecular Probes, Eugene, OR) for 1 hour. Nuclei were counterstained with 4′,6-diamidino-2-phenylindole (0.05 mg/ml). Labeled cells were observed under a fluorescence microscope (BZ-9000E; Keyence, Osaka, Japan), and images were obtained using a BZ-II analyzer (Keyence, Osaka, Japan).

### Flow cytometric analysis

Subconfluent cells were harvested by exposure to trypsin/EDTA for 5 minutes at 37°C, and then washed in DMEM/10% FBS. The cells were resuspended in 1% FBS/PBS at 1 × 10^6^ cells/95 μl and incubated with 5 μl phycoerythrin (PE)-conjugated anti-PDPN antibody (12–9381; eBioscience Inc., San Diego, CA) on ice for 30 minutes. We also stained cells with nonspecific rat IgG2 Control PE (12–4321; eBioscience Inc.) for the negative control. To detect FAP positive cells, the cells were resuspended in 1% FBS/PBS at 1 × 10^6^ cells/95 μl and incubated with 5 μl (2.5 μg) anti-human FAP mouse monoclonal antibody (MAB3715; R&D) on ice for 30 minutes. After washing twice, the cells in 99 μl of 1% FBS/PBS were incubated with 1 μl of allophycocyanin (APC) anti-mouse IgG antibodies (Sony Corporation, Tokyo, Japan) on ice for 30 minutes. Labeled cells were analyzed using a flow cytometer (EC800; Sony) equipped with a laser that provided an excitation wavelength of 488 nm for PE and 642 nm for APC. Data were analyzed using Eclipse Analysis software (Sony).

### Isolation of CAFs by immunoreactivity for PDPN

After labeling the cells with PE-conjugated anti-PDPN antibody, we added magnetic microbeads conjugated with an anti-PE reagent, followed by incubation for 15 minutes at 4°C. PDPN+ cells were isolated by passing the suspension through an AutoMACS PRO separator (Miltenyi Biotechnology). The purity of isolated populations was about 95%. Unlabeled cells were negatively selected and collected by the depletion method through the AutoMACS PRO separator. Unlabeled cells were almost 0% PDPN+.

### Indirect co-culture system

Indirect co-culture was performed using a 6-well transwell culture system with 3-μm cell culture inserts (Becton Dickinson Labware, Franklin Lakes, NJ) as described previously [38]. CAFs were co-cultured with pancreatic cancer cells. Two types of CAFs (5 × 10^4^ cells) were seeded in the lower chambers. After 24 hours of culture, 5 × 10^4^ cells from cancer cell lines were seeded in the upper chambers with DMEM supplemented with 2% FBS. After incubation for 72 and 120 hours, we examined the percentage of PDPN+ CAFs by flow cytometry. For the control, the percentage of PDPN+ CAFs in monoculture was examined.

### Migration and matrigel invasion assays

Migration and invasion of cultured cancer cells were assessed by counting the number of cells migrating or invading through uncoated or Matrigel-coated transwell chambers (BD Biosciences, Franklin Lakes, NJ) as described previously [49,50]. We used transwell inserts with 8-μm pores. Uncoated transwell chambers were used for the migration assay, and chambers coated with 20 μg/well Matrigel (BD Biosciences, Bedford, MA) were used for the invasion assay. Cancer cells (5 × 10^4^ cells/ml, 0.25 ml medium) were seeded in the upper chambers. For co-cultures, 5 × 10^4^ CAFs/0.75 ml medium were seeded in the lower chambers at 24 hours before cancer cell seeding. The cells were then incubated for 18 hours (PANC-1) and 24 hours (SUIT-2) for the migration assay, and 24 hours (PANC-1) and 48 hours (SUIT-2) for the invasion assay. Cancer cells at the lower surface of the membrane were fixed with 70% ethanol, stained with hematoxylin and eosin, and counted in five random fields at × 200 magnification under a microscope. Each experiment was carried out in triplicate wells, and independent experiments were repeated at least three times.

### Silencing of PDPN by siRNA

We performed knockdown of PDPN by siRNA according to the manufacturer’s instructions. CAFs (1 × 10^5^ cells) were transfected with PDPN-1 siRNA (si1) and T1A-2 siRNA (si2) siRNA (Qiagen)/Lipofectamine RNAiMax transfection reagent (Invitrogen)/Opti-MEM (Invitrogen) complexes for the indicated time. After transfection, the cells were cultured in fresh DMEM containing 10% FBS at 37°C. The effect of siRNA was confirmed by qRT-PCR and western blotting. To verify the specificity of knockdown, we used negative control siRNA (Qiagen). CAFs were used in subsequent experiments at 72 hours after transfection.

### Western blotting analysis

Protein was extracted from CAFs using PRO-PREP (iNtRON biotechnology, Seongnam, Korea) according to the manufacturer’s instructions. From each sample, 20 μg of protein was run on a 4% to 12% gradient Bis-Tris–HCl buffered (pH 6.4) polyacrylamide gel (NuPAGE Novex 4–12% Bis-Tris Gel, Invitrogen, Life Technologies, Carlsbad, CA) and transferred to a polyvinylidene difluoride membrane (Millipore, Billerica, MA). The membrane was incubated overnight at 4°C with anti-PDPN (11–003; 1:200; AngioBio Co., Del Mar, CA), anti-CD10 (NCL-CD10-270; 1:100; Novocastra, Newcastle Upon Tyne, UK), or anti-α-tubulin (05–829; 1:1000; Millipore, Billerica, MA) antibodies and then probed with secondary antibodies conjugated to horseradish peroxidase (Santa Cruz Biotechnology). Immunoblots were detected by enhanced chemiluminescence with ChemiDoc XRS (Bio-Rad Laboratories).

### Statistical analysis

Values were expressed as the mean ± standard deviation. Comparisons between two groups were made using the Student’s *t*-test. All experiments were repeated three times. Statistical significance was defined as *P* < 0.05. The χ^2^ test was used to analyze correlations between immunohistochemical staining of PDPN and clinicopathologic characteristics. Survival curves were constructed using the Kaplan-Meier method and compared using the log-rank test. To evaluate independent prognostic factors associated with survival, a multivariate Cox proportional-hazards regression analysis was used. All statistical analyses were performed using JMP 8.0 software (SAS Institute, Cary, NC).

## Competing interests

The authors declare that they have no competing interests.

## Authors’ contributions

YO formulated the study design. KS and SA designed the experiments, carried out the study, and prepared the manuscript. KO supervised the statistical analysis and revised the manuscript. KF provided clinical data including prognostic markers and survival. MF, YM, and MH performed the histopathological assessments of the patients. KM and MT supervised the interpretations of results, and revised the manuscript. All authors read and approved the final manuscript.

## Supplementary Material

Additional file 1: Figure S1 Flow cytometric analysis and immunocytochemical staining of CAFs to characterize. **(A)** The rate of PDPN expression in CAFs did not differ according to the length of trypsin/EDTA treatment after 2, 5, and 10 minutes. **(B)** Immunocytochemical staining of PDPN for cultured CAFs (upper) and harvested CAFs after trypsin/EDTA treatment for 5 min (lower). Original magnification ×400. **(C)** All the primary cultured CAFs (CAF1, CAF2, CAF3, and CAF4) were positive for FAP in most cells. Click here for file

Additional file 2: Figure S2 CAFs (CAF3 and CAF4) were sorted into two populations based on their expression of PDPN to investigate the biological functions of PDPN+ CAFs. **(A)** Analysis of the PDPN+ population among parental CAF4 cells (left) and reanalysis of the sorted PDPN+ cells (right upper, 90.8% PDPN+ cells) and PDPN– cells (right lower, 0.44% PDPN+ cells). **(B)** qRT-PCR was performed to measure the PDPN mRNA expression in CAF1 cells, CAF2 cells, and sorted PDPN+ and PDPN– CAFs. Click here for file

Additional file 3: Figure S3 PDPN knockdown in CAF1 cells by siRNA. Transfection of PDPN-1 siRNA (si1) and T1A-2 siRNA (si2) decreased PDPN mRNA expression **(A)** resulting in decreased levels of PDPN protein in cells as shown by western blotting at the indicated times **(B)**. Knockdown of PDPN in CAF1 cells by siRNA showed no differences in the migration **(C)** or invasion **(D)** of PANC-1 and SUIT-2 cells compared with the control cells (n.s.: not significant). Click here for file

Additional file 4: Table S1 Clinicopathological characteristics of the 105 patients with pancreatic IDC. Click here for file

Additional file 5: Table S2 Primers used for qRT-PCR. Click here for file
