# Supplementary material for: Podoplanin expression in cancer-associated fibroblasts enhances tumor progression of invasive ductal carcinoma of the pancreas
Source: Mol Cancer. 2013 Dec 20;12:168. doi: 10.1186/1476-4598-12-168 (PMC3916072; doi:10.1186/1476-4598-12-168)
Supplement: Additional file 1: Figure S1 — Flow cytometric analysis and immunocytochemical staining of CAFs to characterize. (A) The rate of PDPN expression in CAFs did not differ according to the length of trypsin/EDTA treatment after 2, 5, and 10 minutes. (B) Immunocytochemical staining of PDPN for cultured CAFs (upper) and harvested CAFs after trypsin/EDTA treatment for 5 min (lower). Original magnification ×400. (C) All the primary cultured CAFs (CAF1, CAF2, CAF3, and CAF4) were positive for FAP in most cells. [file 1476-4598-12-168-S1.pptx]

## Slide 1
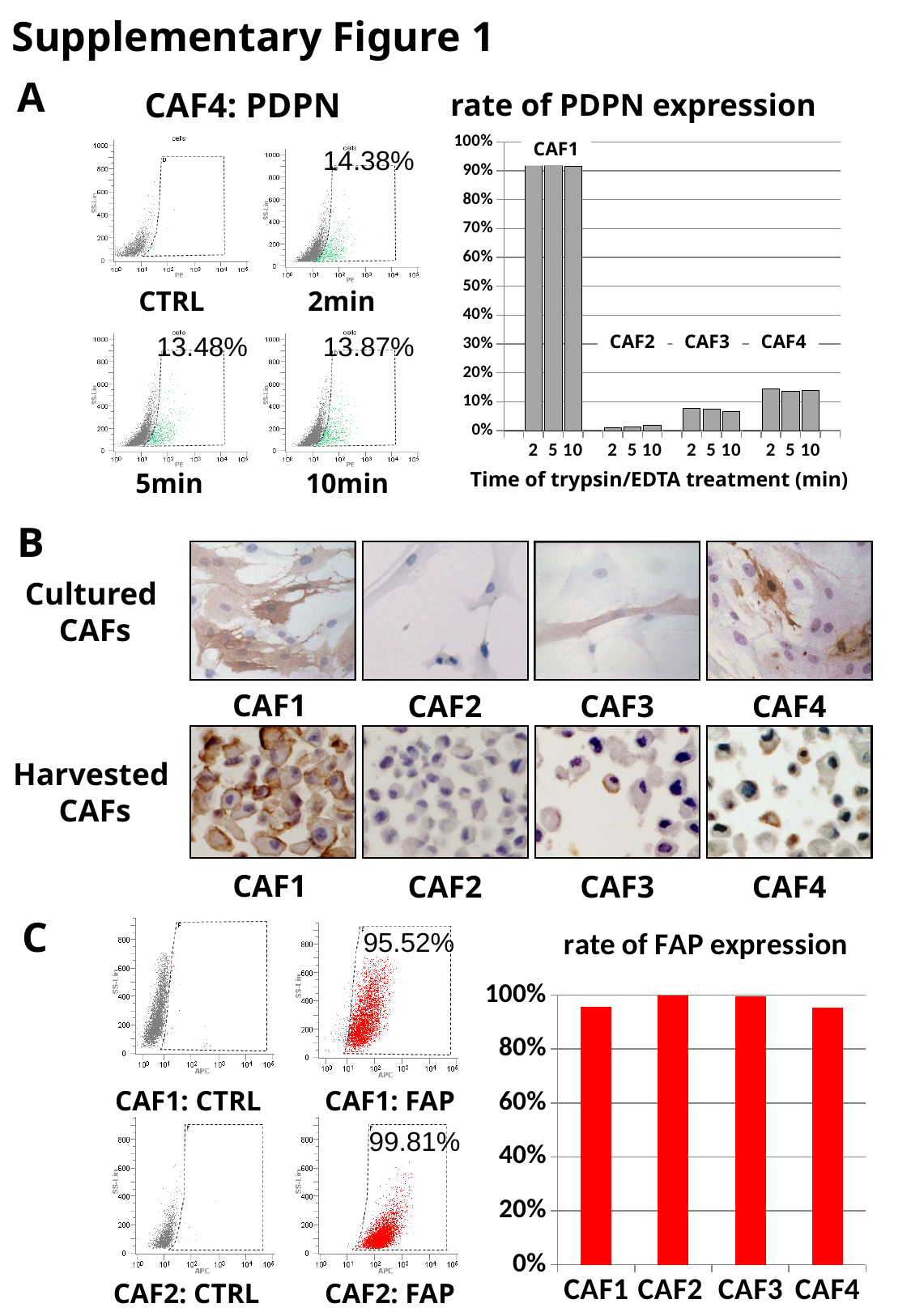

Supplementary Figure 1
A
CAF4: PDPN
14.38%
CTRL
2min
13.87%
5min
10min
13.48%
rate of PDPN expression
### Chart
| Category | CAF1 |
|---|---|
| - | 0.0 |
| 2 | 0.9388598781549173 |
| 5 | 0.9169237549581314 |
| 10 | 0.9158674803836094 |
| | 0.0 |
| 2 | 0.0097 |
| 5 | 0.0121 |
| 10 | 0.0192 |
| | 0.0 |
| 2 | 0.0763 |
| 5 | 0.0739 |
| 10 | 0.0666 |
| | 0.0 |
| 2 | 0.1438 |
| 5 | 0.1348 |
| 10 | 0.1387 |CAF1
CAF2
CAF3
CAF4
Time of trypsin/EDTA treatment (min)
B
Cultured
 CAFs
CAF1
CAF3
CAF4
CAF2
Harvested
 CAFs
CAF1
CAF3
CAF4
CAF2
C
### Chart:
| Category | rate of FAP expression |
|---|---|
| CAF1 | 0.9552 |
| CAF2 | 0.9981 |
| CAF3 | 0.9948 |
| CAF4 | 0.9544 |
95.52%
CAF1: CTRL
CAF1: FAP
99.81%
CAF2: CTRL
CAF2: FAP
