# Supplementary material for: Podoplanin expression in cancer-associated fibroblasts enhances tumor progression of invasive ductal carcinoma of the pancreas
Source: Mol Cancer. 2013 Dec 20;12:168. doi: 10.1186/1476-4598-12-168 (PMC3916072; doi:10.1186/1476-4598-12-168)
Supplement: Additional file 2: Figure S2 — CAFs (CAF3 and CAF4) were sorted into two populations based on their expression of PDPN to investigate the biological functions of PDPN+ CAFs. (A) Analysis of the PDPN+ population among parental CAF4 cells (left) and reanalysis of the sorted PDPN+ cells (right upper, 90.8% PDPN+ cells) and PDPN– cells (right lower, 0.44% PDPN+ cells). (B) qRT-PCR was performed to measure the PDPN mRNA expression in CAF1 cells, CAF2 cells, and sorted PDPN+ and PDPN– CAFs. [file 1476-4598-12-168-S2.pptx]

## Slide 1
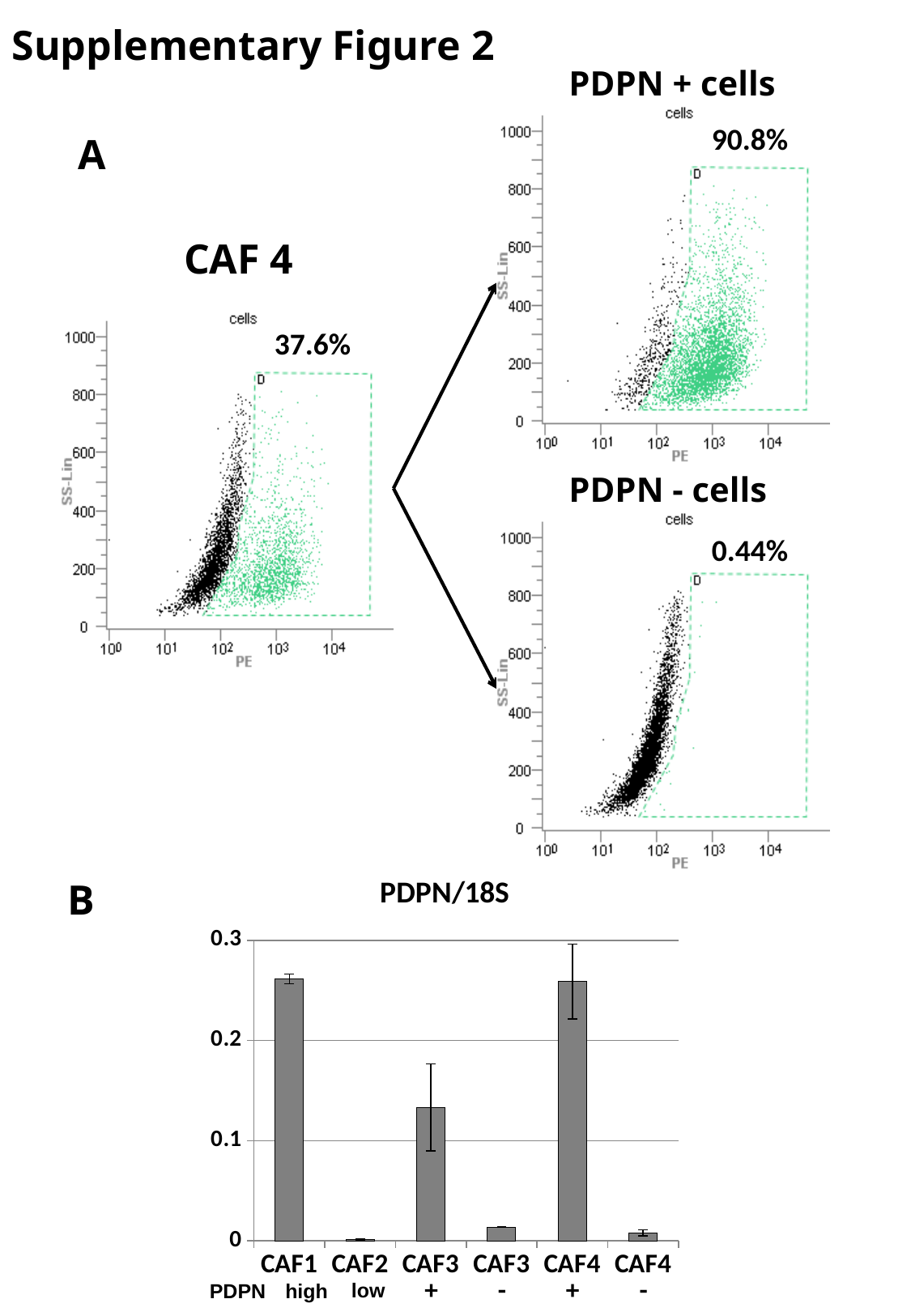

Supplementary Figure 2
PDPN + cells
90.8%
A
CAF 4
37.6%
PDPN - cells
0.44%
B
### Chart: PDPN/18S
| Category | PDPN/18s |
|---|---|
| CAF1 | 0.2615182844897583 |
| CAF2 | 0.001272541379952738 |
| CAF3 + | 0.1331659721476046 |
| CAF3 - | 0.01381762947086013 |
| CAF4 + | 0.258823529411765 |
| CAF4 - | 0.0078518518518518 |low
PDPN
high
