# Supplementary material for: Podoplanin expression in cancer-associated fibroblasts enhances tumor progression of invasive ductal carcinoma of the pancreas
Source: Mol Cancer. 2013 Dec 20;12:168. doi: 10.1186/1476-4598-12-168 (PMC3916072; doi:10.1186/1476-4598-12-168)
Supplement: Additional file 3: Figure S3 — PDPN knockdown in CAF1 cells by siRNA. Transfection of PDPN-1 siRNA (si1) and T1A-2 siRNA (si2) decreased PDPN mRNA expression (A) resulting in decreased levels of PDPN protein in cells as shown by western blotting at the indicated times (B). Knockdown of PDPN in CAF1 cells by siRNA showed no differences in the migration (C) or invasion (D) of PANC-1 and SUIT-2 cells compared with the control cells (n.s.: not significant). [file 1476-4598-12-168-S3.pptx]

## Slide 1
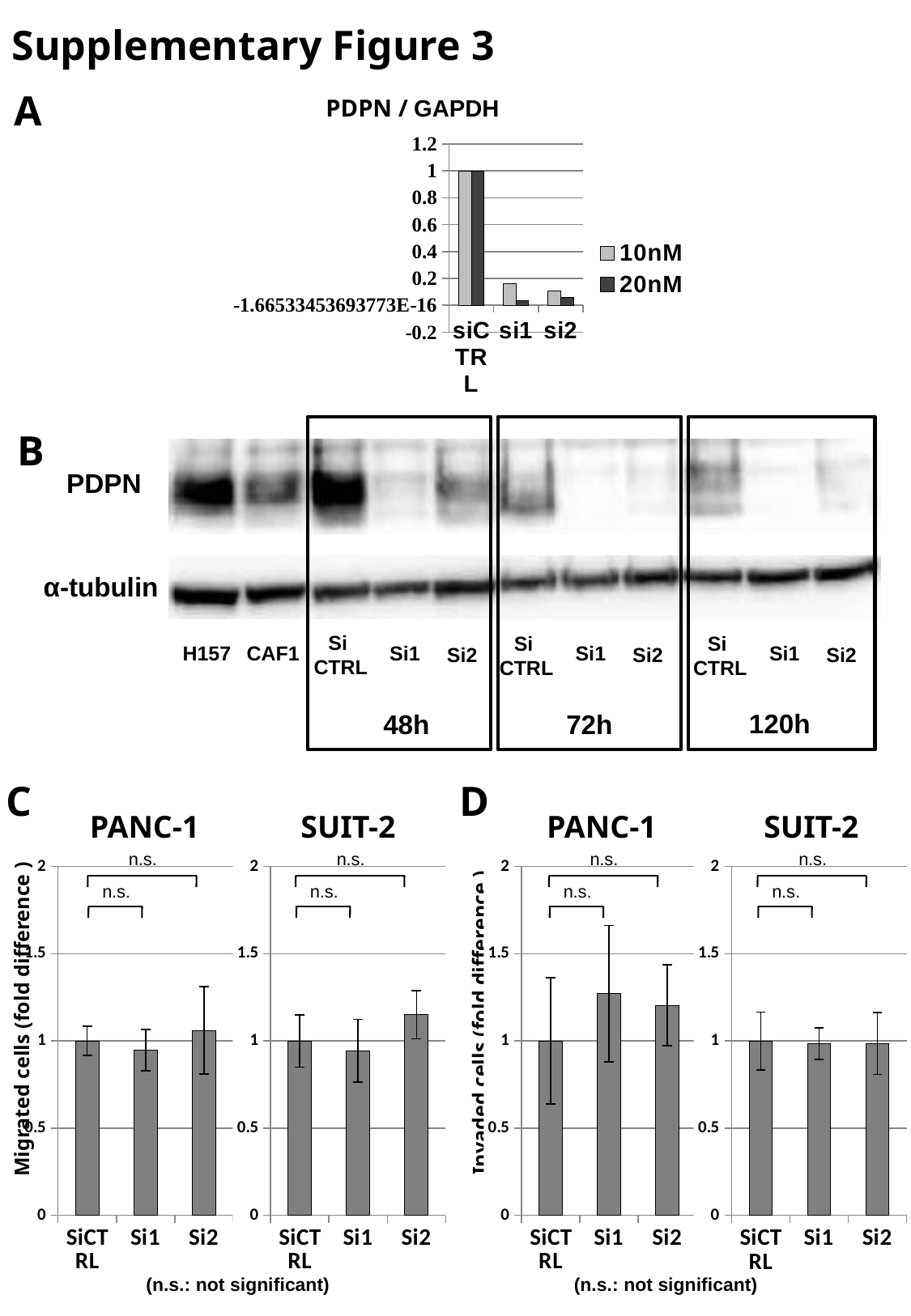

Supplementary Figure 3
A
PDPN / GAPDH
### Chart
| Category | 10nM | 20nM |
|---|---|---|
| siCTRL | 1.0 | 1.0 |
| si1 | 0.16116960910046102 | 0.03651788824026225 |
| si2 | 0.10606447688296249 | 0.0599757889126661 |B
Si
CTRL
Si
CTRL
Si
CTRL
H157
CAF1
Si1
Si1
Si1
Si2
Si2
Si2
120h
48h
72h
PDPN
α-tubulin
C
D
PANC-1
SUIT-2
PANC-1
SUIT-2
n.s.
n.s.
n.s.
n.s.
n.s.
n.s.
n.s.
n.s.
### Chart
| Category | PANC-1 mig |
|---|---|
| SiCTRL | 1.0 |
| Si1 | 0.9469696969696985 |
| Si2 | 1.060606060606062 |
### Chart
| Category | SUIT-2 mig |
|---|---|
| SiCTRL | 1.0 |
| Si1 | 0.9439297903461749 |
| Si2 | 1.1506582155046319 |
### Chart
| Category | PANC-1 inv |
|---|---|
| SiCTRL | 1.0 |
| Si1 | 1.2700729927007326 |
| Si2 | 1.204379562043796 |
### Chart
| Category | SUIT-2 inv |
|---|---|
| SiCTRL | 1.0 |
| Si1 | 0.9839633447880866 |
| Si2 | 0.9853951890034365 |Migrated cells (fold difference )
Invaded cells (fold difference )
(n.s.: not significant)
(n.s.: not significant)
